# Dyadic Adaptation to Moving into a Senior Housing Facility: Changes in Psychological Well-Being

**DOI:** 10.1093/geroni/igaf122.1394

**Published:** 2025-12-31

**Authors:** Gloria Luong

**Affiliations:** Colorado State University, Fort Collins, Colorado, United States

## Abstract

For older adults, moving to a new housing facility (e.g., independent living, assisted living) has been linked to poorer well-being, including greater negative affect, loneliness, and depressive symptoms. Much of this work has focused on individuals, with less research examining how romantic partners navigate this major life event together. In the current study, we investigated how married heterosexual couples (N = 41 couples, 82 individuals) adapted to the transition into housing facilities for older adults. Participants ranged in age from 40-95 years old. Using a prospective longitudinal measurement burst design spanning approximately 3-5 years, participants in the Relocation and Transitional Experiences (RELATE) study completed ecological momentary assessment (EMA) surveys over 8 consecutive days, questionnaires, and health and cognitive assessments at each of 5 bursts. We found that over the 5 bursts, husbands tended to show greater trait positive affect and fewer depressive symptoms than wives. Moreover, husbands showed decreased empathy (perspective taking) over time, compared to wives. Implications for dyadic approaches to developmental transitions in later life are discussed.

